# Narrative Review of Clinical Indices and Imaging Tools, With a Proposal for an Integrative Grading Scale for Vulvovaginal Aging

**DOI:** 10.7759/cureus.96077

**Published:** 2025-11-04

**Authors:** Ana Rodas, Gabriela Sanabria, Pablo González-Isaza

**Affiliations:** 1 Research, Asociación Paraguaya de Medicina Estética (ASOMEDES), Asuncion, PRY; 2 Faculty of Medical Sciences, Universidad Nacional de Asunción, Asuncion, PRY; 3 Urogynecology, San Jorge University Hospital, Pereira, COL

**Keywords:** genitourinary syndrome of menopause, photogrammetry, ultrasound, vaginal atrophy, vulvovaginal aging

## Abstract

Genitourinary syndrome of menopause (GSM) encompasses vulvar and vaginal symptoms and signs related to estrogen deficiency that affect quality of life, sexual function, and urogenital health. This narrative review synthesizes clinical signs, patient-reported outcomes, and commonly used clinician-reported indices to appraise vulvovaginal aging, highlighting strengths and gaps of available tools. Core clinician-reported measures include the Vaginal Health Index (VHI) and the Vulvar Health Index (VuHI), whereas patient-reported measures span global symptom ratings and validated questionnaires such as the Day-to-Day Impact of Vaginal Aging (DIVA). Objective markers (e.g., vaginal pH), cytological indices (vaginal maturation index), and emerging imaging or three-dimensional assessments complement clinical evaluation but remain variably standardized. Across tools, heterogeneity in construct definition, scoring anchors, and responsiveness may hinder comparability and clinical decision-making. We summarize practical, evidence-informed recommendations to improve assessment consistency in routine care and research, and we delineate priorities for future validation, cross-cultural adaptation, and minimal clinically important difference (MCID) determination.

## Introduction and background

The concept of *vaginal aging* has evolved into the more precise term genitourinary syndrome of menopause (GSM), recognizing its impact extends beyond simple atrophy. However, clinical assessment remains fragmented [1]. Whereas in cosmetic dermatology, scales such as the Glogau scale allow for the standardization of diagnosis and treatment of facial photoaging [2], no such analog exists for the female genital area.

Drawing lessons from general cutaneous aging, Galván's work serves as a reminder that this process is a summation of intrinsic (genetics, age) and extrinsic factors (sun exposure, lifestyle) [3]. Although the vulva is not photoexposed, it is subjected to other stressors such as friction, childbirth, and, most significantly, hormonal deprivation. Galván's concepts regarding collagen loss, chronic dehydration, and changes in the extracellular matrix are directly applicable to vulvovaginal tissue and must be considered in any comprehensive assessment scale [3].

Vulvovaginal aging (VVA) integrates hormonal (perimenopausal and postmenopausal hypoestrogenism), anatomical (epithelium, fascia, erectile and adipose tissue), functional (lubrication, pain, laxity), and aesthetic (labial volume, skin texture, and color) changes. The term GSM replaces *vulvovaginal atrophy* and incorporates genital and urinary signs and symptoms attributable to hypoestrogenism, encompassing the vagina, vestibule, vulva, urethra, and bladder. This redefinition drives the need for objective tools and validated scales to characterize VVA beyond isolated symptoms and, notably, to integrate volume, cutaneous quality, and age into a single clinical score useful for treatment indication, follow-up, and outcome assessment [4].

In recent years, key contributions have emerged: (1) specific anatomical-topographical frameworks for the labia majora, highlighting their transitional epithelium, fascia, erectile tissue, and adipose component-structures directly implicated in aesthetic and functional complaints and in the selection of non-surgical technologies; (2) clinical indices parallel to the classic Vaginal Health Index (VHI), such as the Vulvar Health Index (VuHI); and (3) imaging metrics (2D-3D transvaginal/introital ultrasound with gel, high-frequency transperineal vulvar ultrasound) and three-dimensional (3D) photogrammetry to quantify volume and tissue thicknesses.

It has also been emphasized that the labia majora region possesses fat and connective tissue compartments with differential aging (atrophy of subcutaneous fat and dermal changes), which justifies the concurrent measurement of cutaneous thickness and volumetric parameters [5,6]. Recent literature describes standardized ultrasound protocols and reviews of vulvar ultrasonography detailing the location, dimensions, and volume of structures/lesions, and provides evidence of the portability and reproducibility of 3D photogrammetry for assessing subtle changes in the vulva [5]. Further supporting this quantitative approach, the study by Wang et al. [7] introduces a crucial and measurable variable: the integrity of support structures. By measuring skin thickness and fascial distance, volume loss and flaccidity - two of the most visible signs of vulvar aging - can be objectively quantified. This approach effectively shifts the assessment from a subjective to an objective framework.

This article proposes to lay the foundation for creating a *Vulvovaginal Aging Assessment Scale*, a tool designed not only to classify the severity of aging but also to guide interventions, whether hormonal, regenerative, or surgical. To this end, it is essential to review and comprehend all the elements currently used to measure this process.

## Review

Materials and methods

This narrative review was based on targeted searches of PubMed, Scopus, and Web of Science for clinical guidelines, reviews, and original validation studies related to VVA assessment. The aim was to synthesize current clinical, imaging, and patient-reported approaches for evaluating VVA. Priority was given to publications describing validated scales such as the VHI, VuHI, and Day-to-Day Impact of Vaginal Aging (DIVA), as well as studies providing objective measurements through ultrasound, 3D photogrammetry, or laboratory parameters (vaginal pH, Vaginal Maturation Index (VMI)).

Searches combined controlled terms and free-text keywords related to VVA, anatomical structures, and imaging modalities. Representative search strings included: (vulvar OR labia majora OR vestibule OR vulvovaginal) AND (aging OR genitourinary syndrome of menopause) AND (ultrasound OR high-frequency ultrasound OR 3D ultrasound OR MRI OR stereophotogrammetry OR 3D imaging); (Vulvar Health Index OR VuHI OR Vaginal Health Index OR VHI OR DIVA) AND (validation OR reliability)

Articles were selected according to their relevance and contribution to the understanding of VVA. Original studies, reviews, and consensus statements were considered if they described quantitative or qualitative methods for evaluating clinical, structural, or functional changes of the vulvovaginal region in adult women. Reports focusing exclusively on malignancy, reconstructive surgery unrelated to aging, or pediatric populations were excluded.

Information extracted from each source included study objectives, population characteristics, type of assessment (clinical, imaging, or patient-reported), and principal findings. Emphasis was placed on identifying common measurement domains, such as vulvar volume, dermal thickness, mucosal condition, and symptom burden, and their psychometric properties (e.g., reliability, validity, responsiveness).

Given its narrative design, no formal protocol registration or risk-of-bias appraisal was performed. Instead, the review sought to integrate the most relevant and methodologically sound evidence to support the conceptual development of an integrative framework for VVA assessment.

Results

The narrative search of literature published and identified as relevant studies that were categorized into three principal domains: (1) Current Clinical and Symptomatic Assessment Scales, (2) Morphological Evaluation of the Labia Majora, and (3) Objective and Imaging-Based Methodologies.

The characteristics of currently utilized clinical, symptomatic, and functional scales are summarized in Table 1. A principal limitation identified across the included literature is that no existing scale simultaneously integrates objective volumetric parameters (e.g., volume, dermal/adipose thickness) with patient age and clinical signs. This gap highlights a significant unmet need in clinical practice and provides a strong rationale for the development of a novel, integrated scoring system.

**Table 1:** Characteristics of clinical and symptomatic scales for the evaluation of vulvovaginal aging.

Scale/Tool (Reference)	Type of assessment	Parameters evaluated	Primary purpose
Vaginal Health Index (VHI) [8]	Clinical (evaluated by a physician)	Elasticity, fluid volume, pH, epithelial integrity, and moisture.	To quantify the degree of vaginal atrophy, focusing on the hormonal status
Vulvar Health Index (VuHI) [9]	Clinical (evaluated by a physician)	Texture, elasticity, vulvar pigmentation.	To offer an objective metric for vulvar health, analogous to VHI
Visual Analog Scale (VAS) [10]	Symptomatic (reported by patient)	Severity of individual symptoms such as dryness, pain, or itching.	To measure the patient's subjective perception of the intensity of her symptoms
Female Sexual Function Index (FSFI) [11]	Functional (reported by patient)	Multiple domains of sexual response (desire, arousal, lubrication, etc.).	To assess the impact of aging changes on sexual function
DIVA Questionnaire [12]	Quality of Life (reported by patient)	Impact on daily life, body image, and sexual function.	To measure how vaginal aging affects a woman's overall quality of life
Vaginal Maturation Index (VMI) and pH [13]	Laboratory/Clinical	Cell proportions (parabasal, intermediate, superficial) and acidity level.	To assess the degree of estrogenization and aging of the vaginal mucosa

Metrics for the morphological evaluation of the labia majora and the characteristics of objective imaging modalities are detailed in Tables 2, 3, respectively. A consistent finding across studies employing these methods was the documentation of age-associated physiological changes. These typically included a reduction in epithelial thickness, atrophy of subcutaneous fat, increased fascial laxity, and altered tissue vascularization.

**Table 2:** Metrics and parameters for the morphological evaluation of the labia majora.

Morphological parameters	Clinical description	Assessment method/metric	Author/key reference
Volume Loss	Atrophy of adipose tissue in the labia majora, resulting in a *skeletal* appearance and increased prominence of the labia minora.	Volume quantification using 3D imaging and magnetic resonance imaging (MRI), especially in postmenopausal women	Almadori et al.[14]
Texture and Pigmentation	Elastosis (loss of elasticity), fine and coarse wrinkles, pigmentary changes (hyper/hypo), loss of luster, and decreased vascularity.	Dermoscopy and standardized clinical photography to assess changes in the skin surface	Alcazar et al. [15], Rees et al. [16]
Laxity and Topography	Elongation and sagging of the labia minora and clitoral hood. The epithelium, fascia, and adipose tissue are key structures.	Standardized topographic classification and assessment of the anatomical structure	González-Isaza et al. [5]
Structural Integrity	Measurement of dermal thickness and the distance between fascia and adipose tissue as markers of collagen and elastin loss.	High-resolution ultrasound to obtain objective measurements of supporting tissue thickness	Wang et al. [7]
Histological Changes	Epidermal thinning, decreased fibroblast count, and alteration of the extracellular matrix in the skin.	Histological analysis of the cellular and structural components of the skin.	Galván García et al. [3]

**Table 3:** Characteristics of objective and imaging methods.

Imaging method/objective	Main application	Parameters measured/observed	Author/key reference
High-frequency ultrasound (vulvar/transperineal)	Quantify volume loss and laxity of vulvar support structures; identify anatomical planes.	Skin/subcutaneous tissue thickness, distance from fascia to adipose tissue, and visualization of dermis and fascial planes	Wang et al. [7], Montik et al. [17]
3D Photogrammetry/stereophotogrammetry	Measure vulvar soft tissue volume noninvasively and with high reproducibility.	Subtle volumetric changes	Alcázar et al. [15]
Vaginoscopy/colposcopy	Visually inspect the vaginal epithelium under magnification to detect signs of atrophy.	Mucosal thickness, pallor, fragility, and presence of petechiae	Rees et al. [16]
3D transvaginal ultrasound with gel	Evaluate the vaginal wall and correlate its condition with genitourinary syndrome of menopause (GSM).	Vaginal wall thickness	Zulfiqar et al. [18]
Biopsy and histological analysis	Evaluate tissue quality at the microscopic level (considered the gold standard).	Quality and quantity of collagen and elastin, level of vascularization	Ros et al. [19]

Across all domains, the included studies consistently affirmed that VVA is a multifactorial phenomenon with significant repercussions for both function (e.g., dyspareunia, dryness, urinary urgency) and aesthetics (e.g., labial atrophy, rhytids, dyschromia). The evidence demonstrates that a combination of objective imaging (ultrasound/3D), standardized anatomical classification (labia majora), and validated clinical scales (VuHI, VHI) provides a robust foundation for designing a comprehensive, integrative assessment tool. A synthesis of the primary methods for evaluating VVA review is presented in Table 4.

**Table 4:** Summary of methods for assessing vulvo-vaginal aging. VuHI, Vulvar Health Index; US, ultrasound; ICC, intraclass correlation coefficient; GSM, genitourinary syndrome of menopause

Domain	Main method	Parameters	Reported reliability	References
Anatomical morphology	Topographic classification (labia majora)	Epithelial, fascia, and adipose compartments	Expert consensus	González-Isaza et al. [5]
Vulvar quality	VuHI (2024)	Texture, pigmentation, elasticity	Preliminary validation	Cucinella et al. [9]
Volume	3D photogrammetry/stereophotogrammetry	Labia volume (mL, mm³)	ICC >0.90 on face/vulva	Almadori et al. [14]
Vulvar thickness	High-frequency transperineal US	Dermis, fat, and fascia (mm)	Descriptive, reproducible	Montik et al. [17]
Vaginal thickness	3D US with gel	Vaginal wall thickness (mm)	Correlation with GSM	Ros et al. [19]
Symptoms	DIVA questionnaire	Impact on quality of life	Multicenter validation	Huang et al [12], Jang et al. [20]

Based on the above and the review of current assessment parameters, a conceptual grading framework is proposed that integrates the evaluation of vulvar volume and turgor, vaginal mucosal status, functional and psychosocial impact, and includes the assessment of vulvar skin quality together with chronological and hormonal age (Table 5).

**Table 5:** Comprehensive Vulvovaginal Aging Assessment Scale (CVAAS): conceptual framework for clinical and research use (non-validated Instrument) VMI, Vaginal Maturation Index

Scoring	Vulvar volume and turgor	Vulvar skin quality	Vaginal mucosal condition	Chronological and hormonal age	Functional and psychosocial impact
0	Preserved volume, turgid labia majora	Homogeneous skin, without pigmentation or visible wrinkles	Pink, hydrated mucosa, no signs of atrophy	<40 years, no hormonal signs of aging	No symptoms, positive body image, preserved sexual function
1	Slight loss of volume, slight sagging	Mild pigmentation or irregular texture	Slight pallor, decreased spontaneous lubrication	40-55 years, perimenopause	Mild discomfort, changes in lubrication, or desire
2	Moderate atrophy, prominent labia minora due to retraction of the labia majora	Superficial wrinkles, focal hyperpigmentation	Moderate atrophy, fragile mucosa, altered pH	55-65 years, established menopause	Occasional dyspareunia, aesthetic or functional concerns
3	Severe atrophy, structural collapse, loss of anatomical contour	Deep wrinkles, loss of elasticity, extensive dyschromia	Severe atrophy, bleeding on contact, low VMI, pH > 5	>65 years, with clinical signs of hypoestrogenism	Significant sexual dysfunction, emotional, or relational impact

Discussion

VVA is a complex phenomenon integrating structural, cutaneous, and functional changes with both aesthetic and clinical repercussions. Recent literature demonstrates significant progress in characterizing this process through novel imaging technologies, clinical scales, and quality of life questionnaires [7,9,17,19]. However, a fragmentation of methodologies persists, which limits standardization and comparability across studies.

First, structural and volumetric measurements have gained prominence with the development of 3D photogrammetry and portable stereophotogrammetry. These techniques, validated in plastic surgery, have been applied to the vulvar area to evaluate the volume of the labia majora and detect subcutaneous fat atrophy-one of the most representative signs of vulvar aging [15]. Complementing this, González-Isaza et al. proposed a standardized topographical classification of the labia majora in 2024, describing the roles of the epithelium, fascia, erectile tissue, and adipose compartment [5]. This provides a fundamental anatomical framework for understanding morphology and its age-related deterioration.

Second, studies on tissue thickness and quality have demonstrated the utility of high-frequency and transperineal ultrasound for evaluating the dermis, adipose tissue, and fascial planes in the vulvar region [7,15,19]. Likewise, 3D transvaginal ultrasound with gel allows for the quantification of vaginal wall thickness and has shown a correlation with the presence of genitourinary syndrome of menopause [9,15]. These findings reinforce the importance of having objective biomarkers to accompany clinical assessment.

In parallel, available clinical scales present limitations. The VHI remains the reference tool for the vaginal domain, while the recently introduced VuHI expands the assessment to include vulvar texture, pigmentation, and elasticity [8, 9]. Nevertheless, neither of these scales integrates volumetric parameters nor directly correlates with chronological age. Quality of life questionnaires, such as the DIVA, are effective for assessing the functional and sexual impact of genital aging but do not quantify structural changes [12].

The contrast with other areas of aesthetic medicine is illustrative. In facial aging, the Glogau Scale has been established as a clinical standard due to its simplicity and its correlation with age and cutaneous changes [2]. In the vulvovaginal field, the absence of an analogous instrument hinders clinical communication and patient stratification in research. The extrapolation of a Glogau-type model is therefore a logical step, as it would allow for the simultaneous integration of volume, tissue quality, and age, offering a practical and objective classification framework.

From a methodological standpoint, a future VVA scale should possess three essential characteristics: (1) high inter-observer reproducibility, ensuring consistency among aesthetic physicians, gynecologists, and dermatologists; (2) correlation with objective biomarkers, such as high-frequency ultrasound and 3D photogrammetry; and (3) clinical simplicity, allowing for its rapid application in daily practice. For its validation, adherence to the COSMIN (COnsensus-based Standards for the selection of health Measurement INstruments) guidelines is fundamental, including analyses of construct validity, reliability, and responsiveness to change [21].

The clinical implications of an integrative conceptual framework are manifold. In the clinical setting, it would permit the documentation of aging progression and the uniform evaluation of outcomes from regenerative interventions such as platelet-rich plasma, collagen biostimulators, laser, and radiofrequency. In the research domain, it would facilitate the comparison of results across multicenter studies and the definition of homogeneous inclusion criteria. Furthermore, it would offer patients an objective and standardized language to understand their condition and the expected benefits of each treatment.

The available evidence demonstrates significant advances in the measurement of VVA but confirms the need for an integrative conceptual framework adapted to the vulvovaginal region. Such a tool would have a significant impact on clinical practice, research, and the physician-patient relationship, establishing itself as an innovative contribution to the field of aesthetic medicine and physiological aging.

## Conclusions

Assessment of vulvovaginal aging relies on a combination of clinician-reported indices (VHI, VuHI), patient-reported outcomes (DIVA, Female Sexual Function Index (FSFI)), and objective measures such as pH, VMI, and imaging techniques. Despite growing evidence, heterogeneity across tools limits comparability and standardization. Integrating subjective symptoms with objective parameters in a consistent evaluation framework could enhance both clinical decision-making and research reproducibility. Future priorities include cross-cultural adaptation of existing scales, establishment of minimal clinically important differences, and consensus on core outcome sets for vulvovaginal aging.
